# Supplementary material for: Genetic Counselling Needs for Reproductive Genetic Carrier Screening: A Scoping Review
Source: J Pers Med. 2022 Oct 11;12(10):1699. doi: 10.3390/jpm12101699 (PMC9605645; doi:10.3390/jpm12101699)
Supplement: Supplementary file 1 [file jpm-12-01699-s001.zip › jpm-1856823 SM S1 Database search parameters.pdf]

Supplementary Materials File S1—Database Search Parameters

1.1. EMBASE—Subject Headings and Text Words

#	**Query**
1	genetic counseling/ or genetic service/
2	carrier screening.mp.
3	genetic counsellor/ or genetic counselor/ or genetic counselling/ or genetic counseling/ or genetic service.mp.
4	1 or 3
5	reproductive behavior/ or reproductive health/
6	reproductive/ or prenatal/ or antenatal/ or preconception/ or early pregnancy/
7	5 or 6
8	2 and 4 and 7

1.2. Medline—Subject Headings and Text Words

#	**Query**
1	genetic counselling.mp. or Genetic Counseling/
2	genetic counselling/ or genetic counseling/ or genetic counsellor/ or genetic counselor/ or genetic service.mp. [mp=title, abstract, original title, name of substance word, subject heading word, floating sub-heading word, key-word heading word, organism supplementary concept word, protocol supplementary concept word, rare disease supplementary concept word, unique identifier, synonyms]
3	1 or 2
4	carrier screening.mp. or Genetic Carrier Screening/
5	Reproductive Health/ or Reproductive Behavior/
6	Preconception Care/ or Pregnancy/ or preconception.mp.
7	5 or 6
8	3 and 4 and 7

1.3. CINAHL—Subject Headings and Text Words

#	**Query**
1	(MH “Genetic counselling”) OR MH(Genetic Counseling (Iowa NIC)”) OR “genetic counselling or genetic counseling”
2	(MH “Carrier State”) OR (MH “Heterozygote”) OR (MH “Genetic Screening”) OR “carrier screening”
3	(MH “Reproductive Health”) OR (MH “Reproductive Behaviour” OR “reproductive”
4	(MH “Prepregnancy Care”) OR “preconception”
5	“prenatal” OR (MH “Prenatal Care”) OR (MH “Prenatal Care (Iowa NIC)”) OR “early pregnancy” OR “first trimester”
6	3 OR 4 OR 5
7	1 and 2 and 6
