# Supplementary material for: Genetic Counselling Needs for Reproductive Genetic Carrier Screening: A Scoping Review
Source: J Pers Med. 2022 Oct 11;12(10):1699. doi: 10.3390/jpm12101699 (PMC9605645; doi:10.3390/jpm12101699)
Supplement: Supplementary file 1 [file jpm-12-01699-s001.zip › jpm-1856823 SM S2 Articles included in scoping review.pdf]

Supplementary Materials S2—Articles Included in Scoping Review Analysis

1. Briggs A, Nouri PK, Galloway M, O'Leary K, Pereira N, Lindheim SR. Expanded carrier screening: a current survey of physician utilization and attitudes. *J Assist Reprod Genet*. 2018;35(9):1631-1640. doi:10.1007/s10815-018-1272-8.
2. Cho D, McGowan ML, Metcalfe J, Sharp RR. Expanded carrier screening in reproductive healthcare: Perspectives from genetics professionals. *Human Reproduction*. 2013;28(6):1725-1730. doi:10.1093/humrep/det091.
3. Conijn T, Nijmeijer SCM, Lakeman P, Henneman L, Wijburg FA, Haverman L. Preconception expanded carrier screening: Impact of information presented by text or video on genetic knowledge and attitudes. *J Genet Couns*. 2021;30(2):457-469. doi:https://dx.doi.org/10.1002/jgc4.1332.
4. Gabriel MC, Rice SM, Sloan JL, Mossayebi MH, Venditti CP, Al-Kouatly HB. Considerations of expanded carrier screening: Lessons learned from combined malonic and methylmalonic aciduria. *Mol Genet Genomic Med*. 2021;9(4):1-4. doi:10.1002/mgg3.1621.
5. Gbur S, Mauney L, Gray KJ, Wilkins-Haug L, Guseh S. Counseling for personal health implications identified during reproductive genetic carrier screening. *Prenat Diagn*. Published online 2021. doi:10.1002/pd.6033.
6. Hardy MW, Kener HJ, Grinzaid KA. Implementation of a Carrier Screening Program in a High-Risk Undergraduate Student Population Using Digital Marketing, Online Education, and Telehealth. *Public Health Genomics*. 2018;21(1-2):67-76. doi:10.1159/000493971.
7. Henneman L, Kooij L, Bouman K, ten Kate LP. Personal experiences of cystic fibrosis (CF) carrier couples prospectively identified in CF families. *Am J Med Genet*. 2002;110(4):324-331. http://ovidsp.ovid.com/ovidweb.cgi?T=JS&PAGE=reference&D=med4&NEWS=N&AN=12116205.
8. Hernandez-Nieto C, Alkon-Meadows T, Lee J, et al. Expanded carrier screening for preconception reproductive risk assessment: Prevalence of carrier status in a Mexican population. *Prenat Diagn*. 2020;40(5):635-643. doi:10.1002/pd.5656.
9. Ioannou L, Massie J, Collins V, McClaren B, Delatycki MB. Population-based genetic screening for cystic fibrosis: attitudes and outcomes. *Public Health Genomics*. 2010;13(7-8):449-456. doi:https://dx.doi.org/10.1159/000276544.
10. Ioannou L, Delatycki M, Massie J, Hodgson J, Lewis S. 'Suddenly Having two Positive People who are Carriers is a Whole New Thing'- Experiences of Couples Both Identified as Carriers of Cystic Fibrosis Through a Population-Based Carrier Screening Program in Australia. *J Genet Couns*. 2015;24(6):987-1000. doi:10.1007/s10897-015-9833-9.
11. Janssens S, Chokoshvili D, Vears DF, de Paepe A, Borry P. Pre- and post-testing counseling considerations for the provision of expanded carrier screening: exploration of European geneticists' views. *BMC Med Ethics*. 2017;18:1-10. doi:10.1186/s12910-017-0206-9.
12. Jelin AC, Anderson B, Wilkins-Haug L, Schulkin J. Obstetrician and gynecologists' population-based screening practices. *Journal of Maternal-Fetal & Neonatal Medicine*. 2016;29(6):875-879. doi:10.3109/14767058.2015.1029910.
13. Johansen Taber KA, Beauchamp KA, Lazarin GA, Muzzey D, Arjunan A, Goldberg JD. Clinical utility of expanded carrier screening: results-guided actionability and outcomes. *GENETICS in MEDICINE*. Published online 2019:21. doi:10.1038/s41436.
14. Jung U, Urner U, Grade K, Coutelle C. Acceptability of carrier screening for cystic fibrosis during pregnancy in a German population. *Hum Genet*. 1994;94(1):19-24. http://ovidsp.ovid.com/ovidweb.cgi?T=JS&PAGE=reference&D=med3&NEWS=N&AN=8034290.
15. Kalfoglou AL, Broder M. Orthodox Ashkenazi Young Adults' Knowledge, Experiences, Attitudes, and Beliefs About Genetic Carrier Testing. *AJOB Prim Res*. 2011;2(2):1-7. doi:10.1080/21507716.2011.600749.
16. Kraft SA, Schneider JL, Leo MC, et al. Patient actions and reactions after receiving negative results from expanded carrier screening. *Clin Genet*. 2018;93(5):962-971. doi:https://dx.doi.org/10.1111/cge.13206.
17. Larsen D, Ma J, Strassberg M, Ramakrishnan R, van den Veyver IB. The uptake of pan-ethnic expanded carrier screening is higher when offered during preconception or early prenatal genetic counseling. *Prenat Diagn*. 2019;39(4):319-323. doi:10.1002/pd.5434.
18. Lazarin GA, Detweiler S, Nazareth SB, Ashkinadze E. Genetic Counselors' Perspectives and Practices Regarding Expanded Carrier Screening after Initial Clinical Availability. *J Genet Couns*. 2016;25(2):395-404. doi:10.1007/s10897-015-9881-1.
19. Lynch FL, Himes P, Gilmore MJ, et al. Time Costs for Genetic Counseling in Preconception Carrier Screening with Genome Sequencing. *J Genet Couns*. 2018;27(4):823-833. doi:10.1007/s10897-017-0205-5.
20. Massie J, Petrou V, Forbes R, et al. Population-based carrier screening for cystic fibrosis in Victoria: the first three years experience. *Aust N Z J Obstet Gynaecol*. 2009;49(5):484-489. doi:https://dx.doi.org/10.1111/j.1479-828X.2009.01045.x.

21. Mathijssen IB, Holtkamp KCA, Ottenheim CPE, et al. Preconception carrier screening for multiple disorders: evaluation of a screening offer in a Dutch founder population. *Eur J Hum Genet*. 2018;26(2):166-175. doi:https://dx.doi.org/10.1038/s41431-017-0056-4.
22. Nesbit CB, Pollack CC, Mascia NS, et al. Interest in and uptake of genetic counseling for preconception carrier screening when offered to predominantly white reproductive-age persons seeking gynecologic care at a single U.S. academic medical center. *J Genet Couns*. 2021;00:1-11. doi:10.1002/jgc4.1457.
23. Nijmeijer SCM, Conijn T, Lakeman P, Henneman L, Wijburg FA, Haverman L. Attitudes of the general population towards preconception expanded carrier screening for autosomal recessive disorders including inborn errors of metabolism. *Mol Genet Metab*. 2019;126(1):14-22. doi:10.1016/j.ymgme.2018.12.004.
24. Nijmeijer SCM, Conijn • Thirsa, Lakeman P, et al. Attitudes of relatives of mucopolysaccharidosis type III patients toward preconception expanded carrier screening. *European Journal of Human Genetics*. 2020;28:1331-1340. doi:10.1038/s41431-020-0648-2.
25. Ong R, Howting D, Rea A, et al. Measuring the impact of genetic knowledge on intentions and attitudes of the community towards expanded preconception carrier screening. *J Med Genet*. 2018;55(11):744-752. doi:10.1136/JMEDGENET-2018-105362.
26. Peyser A, Singer T, Mullin C, et al. Comparing ethnicity-based and expanded carrier screening methods at a single fertility center reveals significant differences in carrier rates and carrier couple rates. *Genet Med*. 2019;21(6):1400-1406. doi:https://dx.doi.org/10.1038/s41436-018-0331-y.
27. Propst L, Connor G, Hinton M, Poorvu T, Dungan J. Pregnant Women's Perspectives on Expanded Carrier Screening. *J Genet Couns*. 2018;27(5):1148-1156. doi:10.1007/s10897-018-0232-x.
28. Rothwell E, Johnson E, Mathiesen A, et al. Experiences among Women with Positive Prenatal Expanded Carrier Screening Results. *J Genet Couns*. 2017;26(4):690-696. doi:10.1007/s10897-016-0037-8.
29. Schneider JL, Goddard KAB, Davis J, et al. "Is It Worth Knowing?" Focus Group Participants' Perceived Utility of Genomic Preconception Carrier Screening. *J Genet Couns*. 2016;25(1):135-145. doi:https://dx.doi.org/10.1007/s10897-015-9851-7.
30. Shapiro AJ, Kroener L, Quinn MM. Expanded carrier screening for recessively inherited disorders: economic burden and factors in decision-making when one individual in a couple is identified as a carrier. *J Assist Reprod Genet*. 2021;38(4):957-963. doi:10.1007/s10815-021-02084-6.
31. Su YN, Hung CC, Lin SY, et al. Carrier screening for spinal muscular atrophy (SMA) in 107,611 pregnant women during the period 2005-2009: a prospective population-based cohort study. *PLoS One*. 2011;6(2):e17067. doi:https://dx.doi.org/10.1371/journal.pone.0017067.
32. Thain E, Shuman C, Miller K, et al. Prenatal and preconception genetic counseling for consanguinity: Consanguineous couples' expectations, experiences, and perspectives. *J Genet Couns*. 2019;28(5):982-992. doi:10.1002/jgc4.1150.
33. Thompson J, Vogel Postula K, Wong K, Spencer S. Prenatal genetic counselors' practices and confidence level when counseling on cancer risk identified on expanded carrier screening. *J Genet Couns*. 2019;28(4):908-914. doi:10.1002/jgc4.1118.
34. Tsianakas V, Atkin K, Calnan MW, Dormandy E, Marteau TM. Offering antenatal sickle cell and thalassaemia screening to pregnant women in primary care: A qualitative study of women's experiences and expectations of participation. *Health Expectations*. 2012;15(2):115-125. doi:10.1111/j.1369-7625.2011.00669.x.
35. van Dijke I, Lakeman P, Sabiri N, et al. Couples' experiences with expanded carrier screening: evaluation of a university hospital screening offer. *European Journal of Human Genetics*. 2021;(May):1-7. doi:10.1038/s41431-021-00923-9.
36. van Steijvoort E, Demuynck R, Peeters H, et al. Reasons affecting the uptake of reproductive genetic carrier screening among nonpregnant reproductive-aged women in Flanders (Belgium). *J Genet Couns*. 2022;00:1-11. doi:10.1002/jgc4.1575.
37. van Steijvoort E, Devolder H, Geysen I, et al. Knowledge, attitudes and preferences regarding reproductive genetic carrier screening among reproductive-aged men and women in Flanders (Belgium). doi:10.1038/s41431-022-01082-1.
