# Supplementary material for: Genetic Counselling Needs for Reproductive Genetic Carrier Screening: A Scoping Review
Source: J Pers Med. 2022 Oct 11;12(10):1699. doi: 10.3390/jpm12101699 (PMC9605645; doi:10.3390/jpm12101699)
Supplement: Supplementary file 1 [file jpm-12-01699-s001.zip › jpm-1856823 SM S3 Genetic counselling outcomes by THEME.pdf]

Supplementary Materials S3—Genetic Counselling Outcomes by THEME

The OFFER of RGCS:

**Outcome**	**Prosp. user studies N (%)**	**Retrosp. user studies N (%)**	**Genetic HCPs N (%)**	**Other HCPs N (%)**	**Total studies N (%)**
Couple-based screening streamlines RGCS counselling	-	1 (4%)	1 (17%)	-	2 (5%)
Preconception offer preferable	1 (17%)	11 (48%)	2 (33%)	1 (33%)	14 (38%)
Individual carrier screening problematic (partner refusal/incomplete information, etc.)	-	1 (4%)	1 (17%)	-	2 (5%)
**TOTAL**	1	13	4	1	19
**PERCENTAGE**	5.3%	68.4%	21.0%	5.3%	100%

INFORMATION:

**Outcome**	**Prosp. user studies N (%)**	**Retrosp. user studies N (%)**	**Genetic HCPs N (%)**	**Other HCPs N (%)**	**Total studies N (%)**
Result uncertainty/clinical significance/clinical utility (variants/genes/conditions)	-	2 (9%)	4 (67%)	2 (67%)	7 (19%)
Importance of education and training programs for HCPs	2 (33%)	7 (30%)	5 (83%)	2 (67%)	15 (41%)
Limitations of RGCS should be included in pre/post-test counselling	1 (17%)	9 (39%)	2 (33%)	-	12 (32%)
Identify difference between RGCS and NIPT /ultrasound	-	1 (4%)	1 (17%)	-	2 (5%)
Negative RGCS does not guarantee risk free pregnancy/healthy baby	-	4 (17%)	1 (17%)	-	5 (14%)
Sensitivity to reproductive options available/accessible with respect to couple and condition	1 (17%)	9 (39%)	3 (50%)	1 (33%)	13 (35%)
Emphasis/importance of personal health implications for carriers	1 (17%)	2 (9%)	2 (33%)	-	5 (14%)
Public education regarding RGCS	3 (50%)	6 (26%)	-	-	9 (24%)
Thorough post-test counselling for carriers/at-risk	1 (17%)	11 (48%)	4 (67%)	-	16 (43%)
Meet with relevant clinician regarding outcome/phenotype	-	1 (4%)	1 (17%)	-	2 (5%)
Thorough/adequate/consistent pre-test counselling/information	2 (33%)	13 (48%)	3 (50%)	-	16 (43%)
Accessibility to support group/patient families	-	-	1 (1%)	-	1 (3%)
RGCS results require careful, consistent, thorough research	-	-	3 (50%)	-	3 (8%)
**TOTAL**	11	63	30	5	109
**PERCENTAGE**	10%	58%	27%	5%	100%

WHO is offering RGCS:

Outcome	Prosp. user studies N (%)	Retrosp. user studies N (%)	Genetic HCPs N (%)	Other HCPs N (%)	Total studies N (%)
Lack of confidence and knowledge in HCPs offering RGCS	-	-	2 (33%)	1 (33%)	3 (8%)
Importance of/underutilisation of referral to genetic counsellors for at-risk/carrier couples/complex carriers	-	8 (35%)	5 (83%)	2 (67%)	14 (38%)
Importance of education and training programs for HCPs	2 (33%)	7 (30%)	5 (83%)	2 (67%)	15 (41%)
Text/video/alternative to pre-test counselling	4 (67%)	11 (48%)	2 (33%)	1 (33%)	17 (46%)
Alternative method for post-test counselling (e.g. telehealth, video)	-	5 (22%)	1 (17%)	1 (3%)	6 (16%)
Attitude of offering HCP influences uptake/reproductive decisions. Neutral language and non-directiveness	-	7 (30%)	-	-	7 (19%)
Genetic HCPs involved in development of education and support resources/tools for RGCS	1 (17%)	6 (26%)	4 (67%)	-	11 (30%)
**TOTAL**	7	44	19	7	77
**PERCENTAGE**	9%	57%	25%	9%	100%

PERSONALISATION:

Outcome	Prosp. user studies N (%)	Retrosp. user studies N (%)	Genetic HCPs N (%)	Other HCPs N (%)	Total studies N (%)
Individualised pre-test counselling for individuals/couples	4 (67%)	10 (43%)	3 (50%)	1 (33%)	17 (46%)
Sensitivity to reproductive options available/accessible with respect to couple and condition	1 (17%)	9 (39%)	3 (50%)	1 (33%)	13 (35%)
Comprehension of results/risk complicated lived experience	1 (17%)	6 (26%)	-	-	7 (19%)
Result delivery should be tailored/agreed upon plan	-	3 (13%)	2 (33%)	1 (33%)	5 (14%)
Consideration of patient/couple beliefs and values	1 (17%)	6 (26%)	1 (17%)	1 (33%)	8 (22%)
Individual carrier screening problematic (partner refusal/incomplete information, etc.)	-	1 (4%)	1 (17%)	-	2 (5%)
Negative results provide reassurance, relief, confidence	-	1 (4%)	-	-	1 (4%)
**TOTAL**	7	36	10	4	57
**PERCENTAGE**	12.3%	63.2%	17.5%	7.0%	100%
